# Unraveling the role of cAMP signaling in *Giardia*: insights into PKA-mediated regulation of encystation and subcellular interactions

**DOI:** 10.1128/msphere.00723-24

**Published:** 2024-10-30

**Authors:** Han-Wei Shih, Germain C. M. Alas, Alexander R. Paredez

**Affiliations:** 1 Division of Biological Sciences, University of Washington, Bothell, Washington, USA; 2 Department of Biology, University of Washington, Seattle, Washington, USA; University of California, Davis, Davis, California, USA

**Keywords:** encystationc, PKA, cyclic AMP

## Abstract

cAMP plays an important role as a second messenger in the stage transition of various protozoan parasites. This signaling pathway relies on multiple effectors, such as protein kinase A (PKA), exchange protein activated by cAMP, and cAMP-response element binding protein transcription factors, to initiate signal transduction in humans. The *Giardia* genome only contains two adenylate cyclases (ACs), a single phosphodiesterase (PDE) and a single known PKA effector, and the specific functions of these components are not fully understood. In our previous research, we demonstrated the important role of AC2-dependent cAMP signaling in promoting the encystation program. Using the NanoBit technology, we emphasized the significance of AC2-dependent cAMP biosynthesis in regulating the dissociation of the PKA regulatory domain (PKAr) and PKA catalytic domain (PKAc). In this study, our objectives are twofold: first, we used the newly developed Split-Halo to examine subcellular interactions of *Gl*PKAr and *Gl*PKAc in *Giardia*; and second, we investigated whether PKAc regulates encystation-specific proteins. Our findings revealed distinct subcellular locations where *Gl*PKAr and *Gl*PKAc interacted during the trophozoite stage, including the flagella, basal bodies, and cytoplasm. Upon exposure to encystation stimuli, the interaction shifted from the flagella to the cytosol. Knockdown of *Gl*PKAc resulted in the downregulation of encystation-specific genes, leading to the production of fewer viable and water-resistant cysts indicating a role for PKA in the transcriptional regulation of encystation. These discoveries contribute to a deeper understanding of the cAMP signaling pathway and its important role in governing *Giardia*’s encystation process.

**IMPORTANCE:**

The precise timing of interactions and subcellular compartmentation play crucial roles in signal transduction. The co-immunoprecipitation assay (CO-IP) has long been utilized to validate protein–protein interactions; however, CO-IPs lack spatial and temporal resolutions. Our recent study used the NanoBit assay, which showcased the reversible protein–protein interaction between PKAr and PKAc in response to cAMP analogs and encystation stimuli. Expanding on this groundwork, this study employs the Split-Halo assay to uncover the subcellular compartments where the PKAr and PKAc protein–protein interactions take place and respond to encystation stimuli. Taken together, these molecular tools provide spatiotemporal information on the protein–protein interaction, which will be useful in the field.

## OBSERVATION

*Giardia lamblia* is a protozoan parasite and the causative agent of giardiasis, a waterborne diarrheal disease that affects humans and various mammals globally. This parasite has a two-phase life cycle comprising a resilient cyst stage and a replicative trophozoite stage. Cysts are the infectious form that transmits giardiasis. Once cysts are ingested from contaminated water and food sources, they release trophozoites that colonize the small intestine. Bile salts and an alkaline pH within the small intestine play crucial roles in stimulating the process of encystation (1). Eventually, the detached cysts transit to the lower intestine and are expelled for transmission.

cAMP signaling plays an important role in initiating differentiation in *Giardia lamblia*, with protein kinase A (PKA) being the only identified conserved effector in *Giardia* (1). PKA activation relies on the binding of cAMP to a regulatory domain (PKAr), which liberates the catalytic kinase subunit (PKAc), rendering it active. Notably, *Giardia lamblia* has one of the smallest kinomes among eukaryotes (2), with only a single *Gl*PKAr (GL50803_9117) and a single *Gl*PKAc (GL50803_11214). *Gl*PKAr interacts with *Gl*PKAc in a cAMP-dependent manner (3). Interestingly, in the trophozoite stage, *Gl*PKAr and *Gl*PKAc localize to the internal axonemes of the anterior flagella, caudal flagella, and their associated basal bodies consistent with the presence of an A-kinase anchoring protein (3). Studies using a custom rabbit polyclonal antibody to PKA indicate that PKAr levels are diminished after the induction of encystation, while our studies of tagged PKAr show that the protein persists on internal axonemes throughout encystation (1, 3). However, information on whether spatiotemporal dynamics of G*l*PKAr and G*l*PKAc interactions change during encystation in *Giardia* is missing.

In our previous study (1), we introduced the NanoBit assay to characterize the cAMP-dependent reversible interaction of *Gl*PKAr and *Gl*PKAc, providing valuable quantitative evidence of protein–protein interactions. However, while the NanoBit assay offers insights into these interactions, it lacks the ability to provide information on subcellular localization. To address this limitation, we turned to Split-Halo, an imageable reporter proved to be a powerful tool for investigating protein–protein interactions in human and plant cells (4, 5). In this study, we employed Split-Halo to investigate the spatiotemporal interaction between *Gl*PKAr and *Gl*PKAc in response to encystation stimuli. Additionally, we genetically knocked down G*l*PKAc to demonstrate its role in the encystation process.

**Fig 1:**
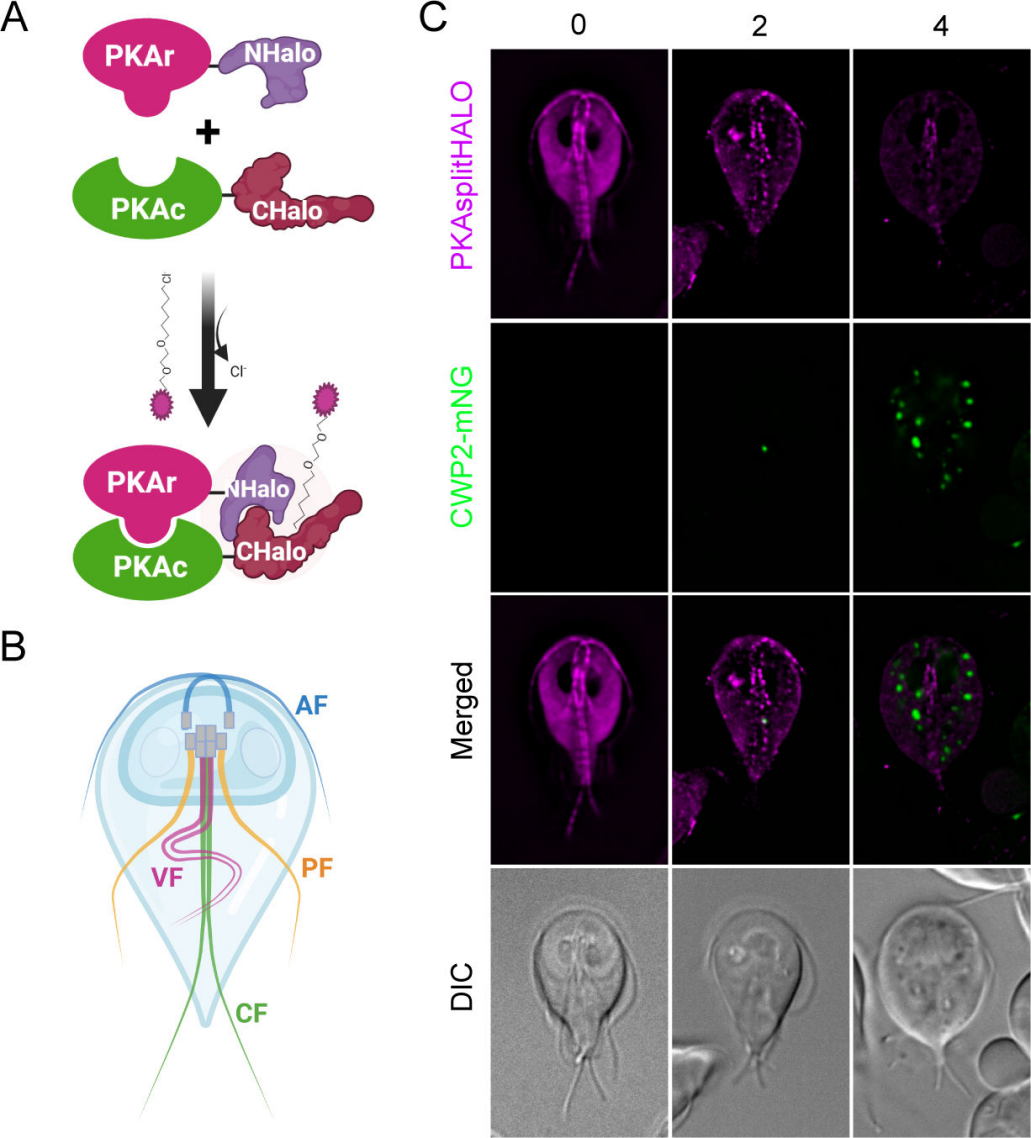
Spatiotemporal interactions of PKA-SplitHalo in response to encystation. A. Schematic of PKA-SplitHalo (GlPKAr-NHalo and GlPKAc-CHalo). Created with BioRender.com. B. Anterior (AF), posteriolateral (PF), caudal (CF), and ventral (VF) flagella are marked in the cyan, yellow, green, and magenta colors. C. Colocalization of PKA-SplitHalo with ESV marker (CWP2-mNG). Colocalization of PKA-SplitHalo and CWP2-mNG (ESV marker) at 0, 2, and 4 h post-exposure to encystation medium; *n* > 20 for each stage. Scale bars, 5 µm.

To investigate protein–protein interactions, split fluorescent proteins have been widely utilized (4). Among these fluorescent proteins, the Halogenase (Halo) tag proved valuable in studying protein subcellular localization due to its low background and the development of its split halo version for investigating protein–protein interactions in human and plant cells (5, 6). Because of the limited success and low fluorescence of the green fluorescent protein under anaerobic conditions, the Halo tag has also been employed as a robust fluorescent reporter for investigating the protein function in anaerobic organisms (7). To precisely examine the subcellular interaction of *Gl*PKAr and *Gl*PKAc, we employed the Split-Halo assay, enabling us to observe the spatiotemporal dynamics of the *Gl*PKAr and *Gl*PKAc interaction in response to encystation stimuli. The *Gl*PKAr protein (*Gl*50803_9117) was tagged with nHalo (1–155), while the *Gl*PKAc protein (GL50803_11214) was tagged with cHalo (156–257). As a control, nHalo (1–155) not fused to any protein was driven by the *Gl*PKAr native promoter (Fig. 1A; Fig. S1A).

Our findings revealed specific subcellular locations where *Gl*PKAr and *Gl*PKAc interact during the trophozoite stage, including the anterior and caudal flagella, basal body, and cytoplasm (Fig. 1B and C; Fig. S1B). CWP2-mNeonGreen (mNG) was used as an encystation marker to observe the spatiotemporal interaction of PKA-Split-Halo in response to encystation stimuli. Upon exposure to encystation stimuli, the interaction at the anterior flagella diminished within 1 h, followed by the disappearance of the interaction at the caudal flagella within 2 h.

In our prior investigation, we revealed that AC2 plays a role in the dissociation of G*l*PKAr and G*l*PKAc, leading to the upregulation of encystation-specific genes like MYB2, CWPs, and enzymes related to GalNAc biosynthesis. However, there is currently no genetic evidence supporting the involvement of *Gl*PKAc in regulating MYB2 and genes associated with cyst wall biosynthesis. To address this, we utilized CRISPRi to generate G*l*PKAc knockdown cell lines (8). The PKAc-g378, PKAc-g664, PKAc-g704, and PKAc-g848 cell lines exhibited reductions of 10, 4, 14, and 35%, respectively, in *Gl*PKAc-NLuc expression (Fig. S2A).

**Fig 2:**
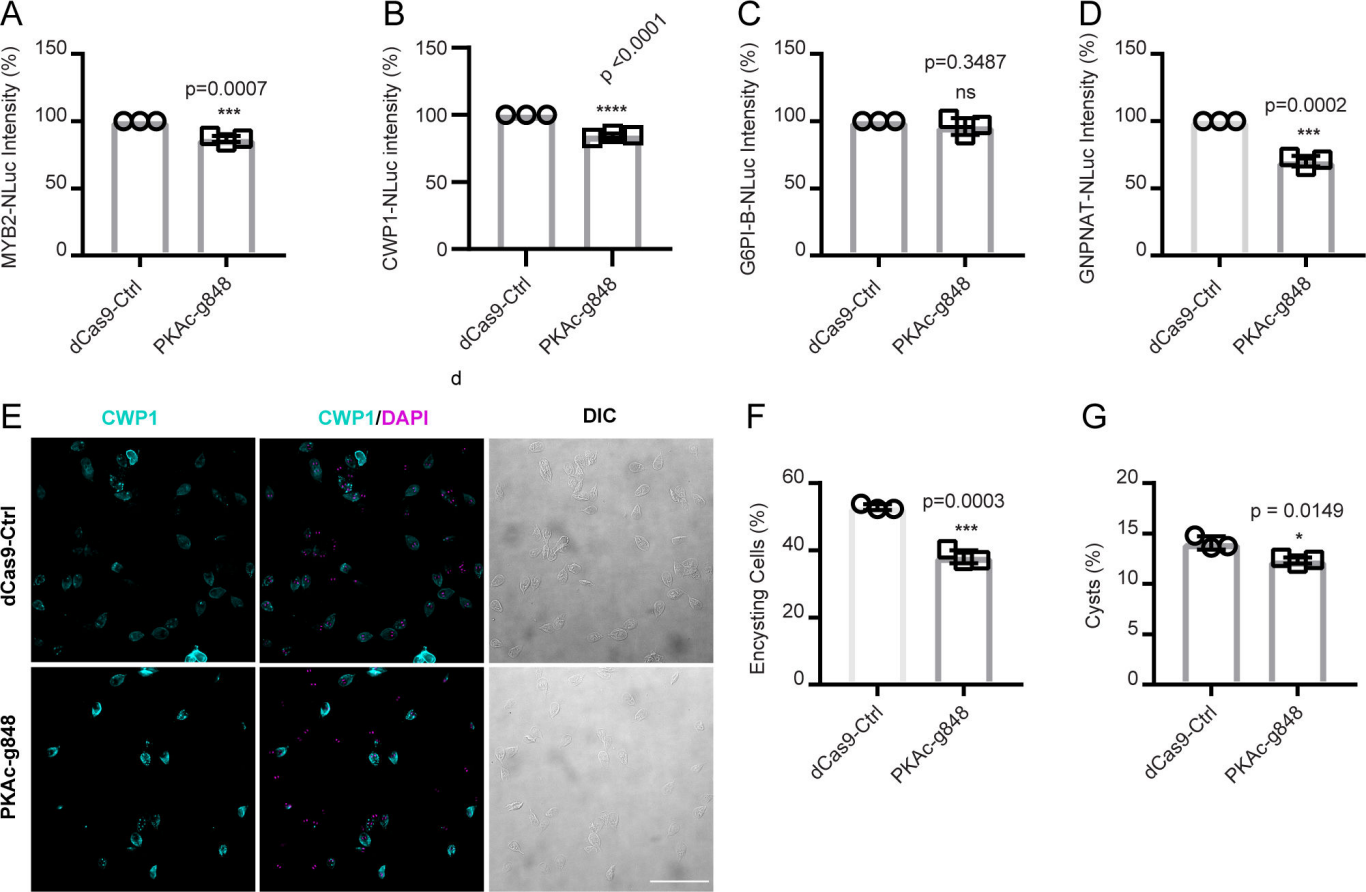
GlPKAc is important for encystation signaling. Relative expression levels of (**A**) MYB2-NLuc (GL50803_8722), (**B**) CWP1-NLuc (GL50803_5638), (**C**) G6PI-B-NLuc (GL50803_8245), and (**D**) GNPNAT-NLuc (GL50803_14259) in dCas9-Ctrl and PKAc-g848 knockdown cell lines. Representative IFA images (**E**) and quantification of 24  h encysting cells (**F**) and cyst (**G**) from dCas9 control and PKAc-g848 knockdown cell lines. Equal numbers of dCas9 control and PKAc-g848 trophozoites (~1.3 × 10^7^) were exposed to encystation medium for 24  h and stained with CWP1 antibody with DAPI (*n* = 1771 for Cas9-ctrl *n*  =  1771 and *n* = 1336 for PKAc-g848). Scale bar, 50  µm. Data are mean  ±  standard deviation from three independent experiments. *P* values were calculated with two-tailed *t*-tests.

We introduced the PKAc guide RNA848 into cell lines expressing encystation-specific genes with NLuc tags, including the master transcription factor (MYB2), cyst wall protein (CWP1), and two GalNAc biosynthesis enzymes (G6PI-B and GNPNAT). Upon exposure to encystation stimuli, PKAc knockdown resulted in a 15.7% decrease in MYB2 expression in the PKAc-g848 cell line (Fig. 2A). Similarly, CWP1 expression decreased by 33.6% in PKAc-g848 cell lines (Fig. 2B). GNPNAT-NLuc expression decreased by 30% after PKAc knockdown (Fig. 2C), while G6PI-B-NLuc expression (Fig. 2D) remained unaffected. Interestingly, our prior study revealed that AC2 knockdown downregulates both GNPNAT and G6PI-B expressions (1), suggesting that the modest reduction of PKAc was insufficient to induce a measurable difference, or that other unidentified cAMP effectors are involved in regulating G6PI-B expression.

Furthermore, PKAc knockdown resulted in 10 and 3% reductions in encysting cells and cysts, respectively, after 24 h of exposure to encystation stimulation (Fig. 2E through G). Additionally, after 48 h of encystation stimuli, followed by 24 h of water incubation, the PKAc knockdown cell line produced fewer water-resistant and viable cysts compared to the dCas9 control (Fig. S2C and D). These findings underscore the crucial role of G*l*PKAc in regulating encystation signal transduction.

Additionally, we investigated the impact of the PKA inhibitor H89 on the expression of CWP1 and the number of encysting cells. Our results demonstrated that pretreatment with H89 for 1 h reduced MYB2 and CWP1 expressions by 40 and 33%, respectively, at 4 h post-encystation stimuli (Fig. S3A and B) but did not affect the number of encysting cells at 24 h (Fig. S3C and D). It is noteworthy that short-term treatment with H89 is limited to 1 h, while long-term exposure is lethal. We interpret this result to indicate that, by 24 h, the treated cells are able to catch up to untreated control cells.

In summary, we employed the Split-Halo assay to investigate subcellular interactions between *Gl*PKAr and *Gl*PKAc in *Giardia*. Spatiotemporal changes in the interaction were observed in response to encystation stimuli, with shifts in cellular locations over time. The CRISPRi-mediated knockdown of *Gl*PKAc resulted in an altered expression of encystation-specific genes, including MYB2, CWP1, and GNPNAT, highlighting *Gl*PKAc’s role in encystation signal transduction and regulation of transcriptional responses to encystation stimuli. This genetic manipulation also led to reduced encysting cell numbers and impaired cyst viability. These findings confirm the utility of Split-Halo for protein–protein interactions and the role of PKA in regulating encystation. Future efforts will focus on identifying the phosphorylation targets of PKA.

### Design of guide RNA for CRISPRi

Guide RNA for the CRISPRi system utilized the Dawson Lab protocol (9). The NGG PAM sequence and *G. lamblia* ATCC 50803 genome were selected for the CRISPRi guide RNA design with Benchling. PKAc-NLuc was created from a previous study. Primers are listed in Table S1.

### Design of GlPKA-Split-Halo

The PKA-NanoBit system and its control, as established in a previous study, were employed to create GlPKA-Split-Halo. In summary, the cHalo fragment (156–257) was PCR-amplified from Halo7 and ligated with PKAc (GL50803_101214) under its native promoter (~500 bp). The nHalo fragment (1–155) was amplified and attached to PKAr (GL50803_9117) also under its native promoter (~500 bp) (Fig. 1A; Fig. S1A). The control cell line expresses PKAc-cHalo (156–257) and the nHalo fragment (1–155) under the native promoter of PKAr (pPKAr) without being fused to any protein (Fig. S1A). Primers are listed in Table S1.

### *Giardia* growth and encystation media

The *Giardia lamblia* isolate WB clone C6 (American Type Culture Collection catalog number 50803;) was cultured in Keister’s modified TYI-S33 media supplemented with 10% adult bovine serum and 0.125 mg/mL bovine bile at pH 7.1. To induce encystation, cells were initially incubated for 48 h in pre-encystation media without bovine bile at pH 6.8, followed by further incubation with media at pH 7.8 supplemented with 10% adult bovine serum, 0.25 mg/mL porcine bile (B8631, Sigma-Aldrich), and 5 mM calcium lactate. H89 inhibitor treatments, specifically N-[2-(p-bromocinnamylamino) ethyl]−5-isoquinolinesulfonamine dihydrochloride (H89, B1427, Sigma-Aldrich), were utilized in this study.

### Live imaging

For live imaging, parasites were released from culture tubes by chilling. They were then transferred to Attoflor imaging chambers that were incubated for 60 min with or without treatments in a Panasonic tri-gas incubator set to 5% CO_2_ and 2% O_2_. To detect HALO tag, 20 nM of Janelia Fluor HaloTag Ligands (Promega, GA112A) was used. Before imaging, parasites were washed with 1× HEPES-buffered saline (HBS). Images were acquired on a DeltaVision Elite microscope using a 60× 1.42-numerical aperture objective with a PCO Edge sCMOS camera, fluorescein isothiocyanate, and Cy5 filter set and deconvolved using SoftWorx (API, Issaquah, WA). For CellMask imaging, CellMask orange plasma membrane stain (Invitrogen C10045) was diluted in a 1:10,000 ratio, incubated for 5 min, and washed three times in 1× HBS.

### *In vitro* bioluminescence assays

*Giardia* cells were chilled on ice for 15 min and then centrifuged at 700×*g* for 7 min at 4°C. Subsequently, the cells were resuspended in cold 1× HBS, and dilutions were prepared after determining cell density using the MOXI Z Mini Automated Cell Counter (Orflo, Kenchum, ID). For the NanoLuc luminescence measurement, 20,000 cells were loaded into white polystyrene, flat-bottom, 96-well plates (Corning Incorporated, Kennebunk, ME) and mixed with 10 µL of the NanoGlo luciferase assay reagent (Promega). Relative luminescence units were detected using a pre-warmed 37°C Perkin Elmer Victor-3 1420 Multilabel Counter for 30 min to achieve the maximum value. These experiments were conducted with three independent bioreplicates.

### Immunofluorescence

*Giardia* parasites were chilled on ice for 30 min and then centrifuged at 700×*g* for 7 min. The resulting pellet was fixed in PME buffer (100 mM piperazine-N,N′-bis(ethanesulfonic acid) (PIPES) pH 7.0, 5 mM EGTA, 10 mM MgSO_4_) supplemented with 1% paraformaldehyde (Electron Microscopy Sciences, Hatfield, PA), 100 µM 3-maleimidobenzoic acid N-hydroxysuccinimide ester (Sigma-Aldrich), 100 µM ethylene glycol bis(succinimidyl succinate) (Pierce), and 0.025% Triton X-100 for 30 min at 37°C. The fixed cells were then attached to polylysine-coated coverslips. After washing once with PME, the cells were permeabilized with 0.1% Triton X-100 in PME for 10 min. Following two quick washes with PME, blocking was carried out in PME supplemented with 1% bovine serum albumin, 0.1% NaN_3_, 100 mM lysine, and 0.5% cold water fish skin gelatin (Sigma-Aldrich). Subsequently, the cells were incubated with a 1:200 dilution of Alexa Fluor 647-conjugated anti-CWP1 antibody (Waterborne, New Orleans, LA) for 1 h. After three washes in PME plus 0.05% Triton X-100, the coverslips were mounted with ProLong Gold antifade plus 4′,6-diamidino-2-phenylindole (DAPI; Molecular Probes). Images were captured using a DeltaVision Elite microscope equipped with a 60× 1.42 numerical aperture objective and a PCO Edge sCMOS camera, followed by deconvolution using SoftWorx (API, Issaquah, WA).

### Image analysis

We employed ImageJ for processing all images, and figures were assembled using Adobe Illustrator.

### Cyst count and cyst viability staining

*Giardia* trophozoites were cultured for 24 h in encystation media supplemented with 10 g/L ovine bovine bile and calcium lactate, followed by another 24 h in TYI-S33 media. After a total of 48 h, the total cell number was determined using the MOXI Z Coulter Counter. The encysted culture was then centrifuged at 700×*g* for 7 min, and the pellets were washed 10 times with deionized water before being stored in distilled water overnight at 4°C. To determine cyst concentration, 20 µL of the 48 h encysted cells were counted using a hemocytometer. Cyst viability was assessed using fluorescein diacetate and propidium iodide staining to differentiate live and dead cysts. Images were captured using a DeltaVision Elite microscope equipped with a 40×, 1.35 numerical aperture objective and a PCO Edge sCMOS camera. The images were subsequently deconvolved using SoftWorx (API, Issaquah, WA).

## SUPPLEMENTAL MATERIAL

Fig. S1 msphere.00723-24-s0001.tif GlPKA Split-Halo construct design.

Fig. S2 msphere.00723-24-s0002.tif PKAc CRISPRi knockdown and cyst viability.

Fig. S3 msphere.00723-24-s0003.tif PKAc inhibition with H89.

Table S1 msphere.00723-24-s0004.docx Oligos used in this study.

ASM does not own the copyrights to Supplemental Material that may be linked to, or accessed through, an article. The authors have granted ASM a non-exclusive, world-wide license to publish the Supplemental Material files. Please contact the corresponding author directly for reuse.
