# Supplementary figures and images for: Unraveling the role of cAMP signaling in Giardia: insights into PKA-mediated regulation of encystation and subcellular interactions
Source: mSphere. 2024 Oct 30;9(11):e00723-24. doi: 10.1128/msphere.00723-24 (PMC11580427; doi:10.1128/msphere.00723-24)

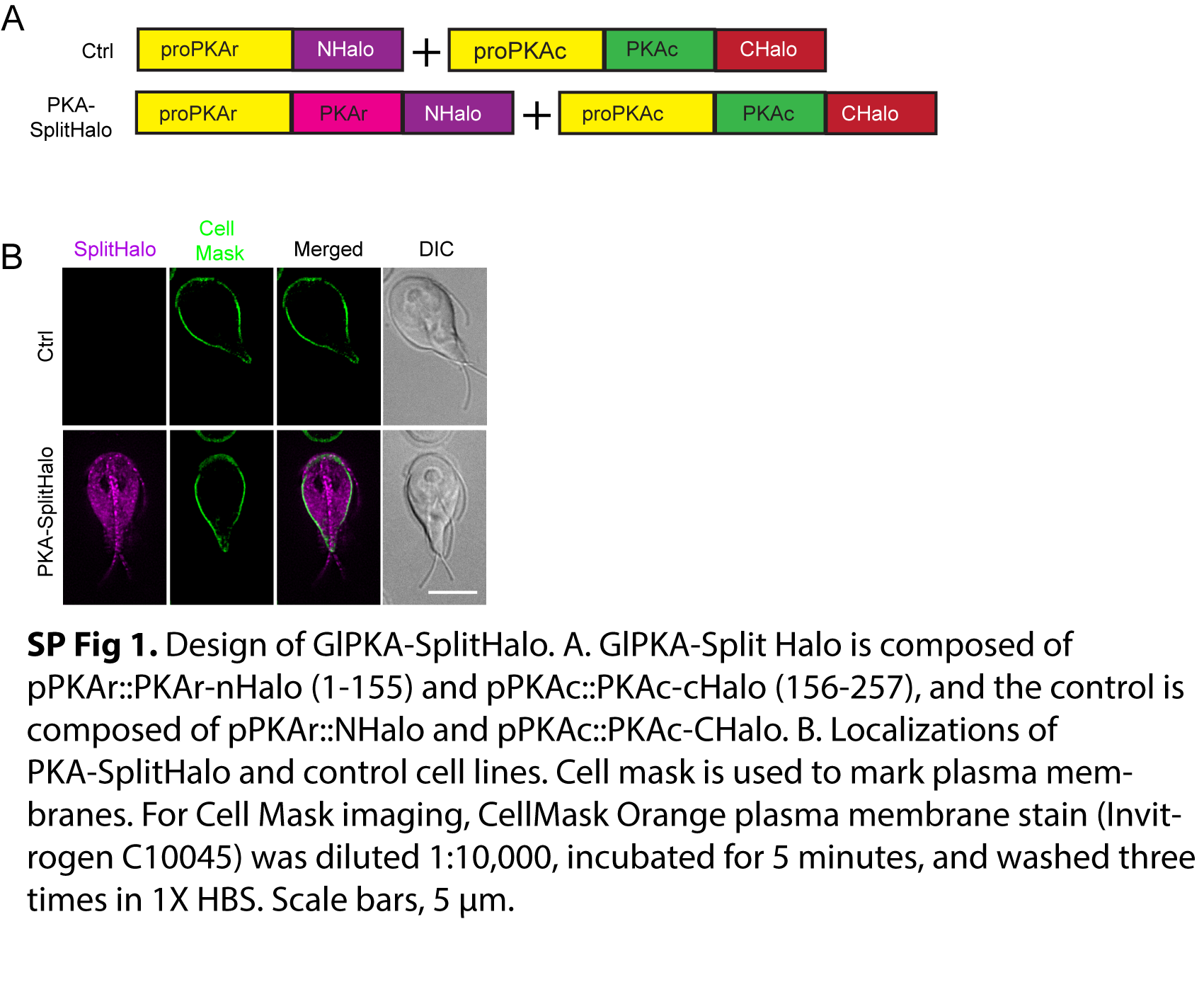

Supplement: Fig. S1 — GlPKA Split-Halo construct design. [file msphere.00723-24-s0001.tif]

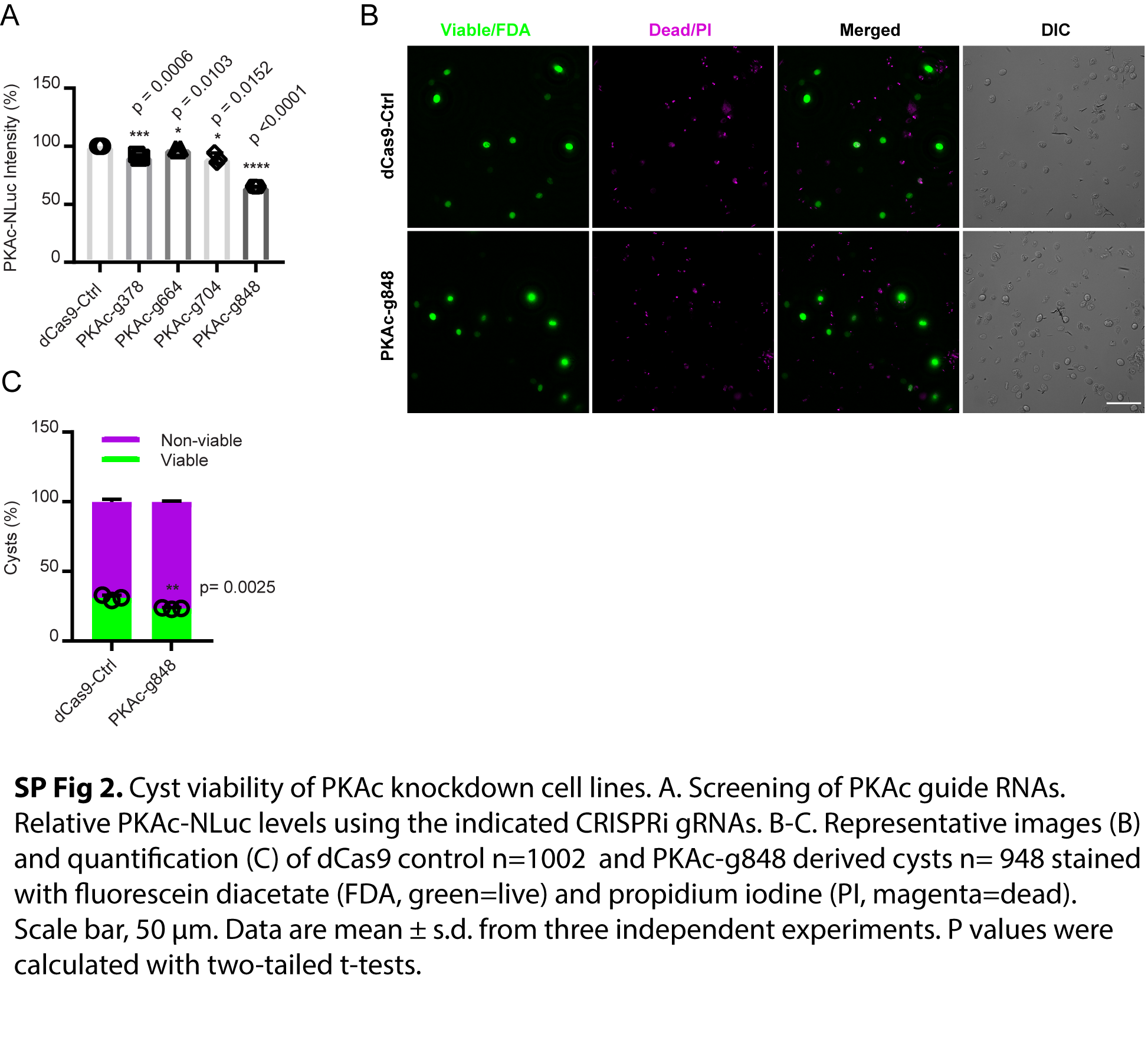

Supplement: Fig. S2 — PKAc CRISPRi knockdown and cyst viability. [file msphere.00723-24-s0002.tif]

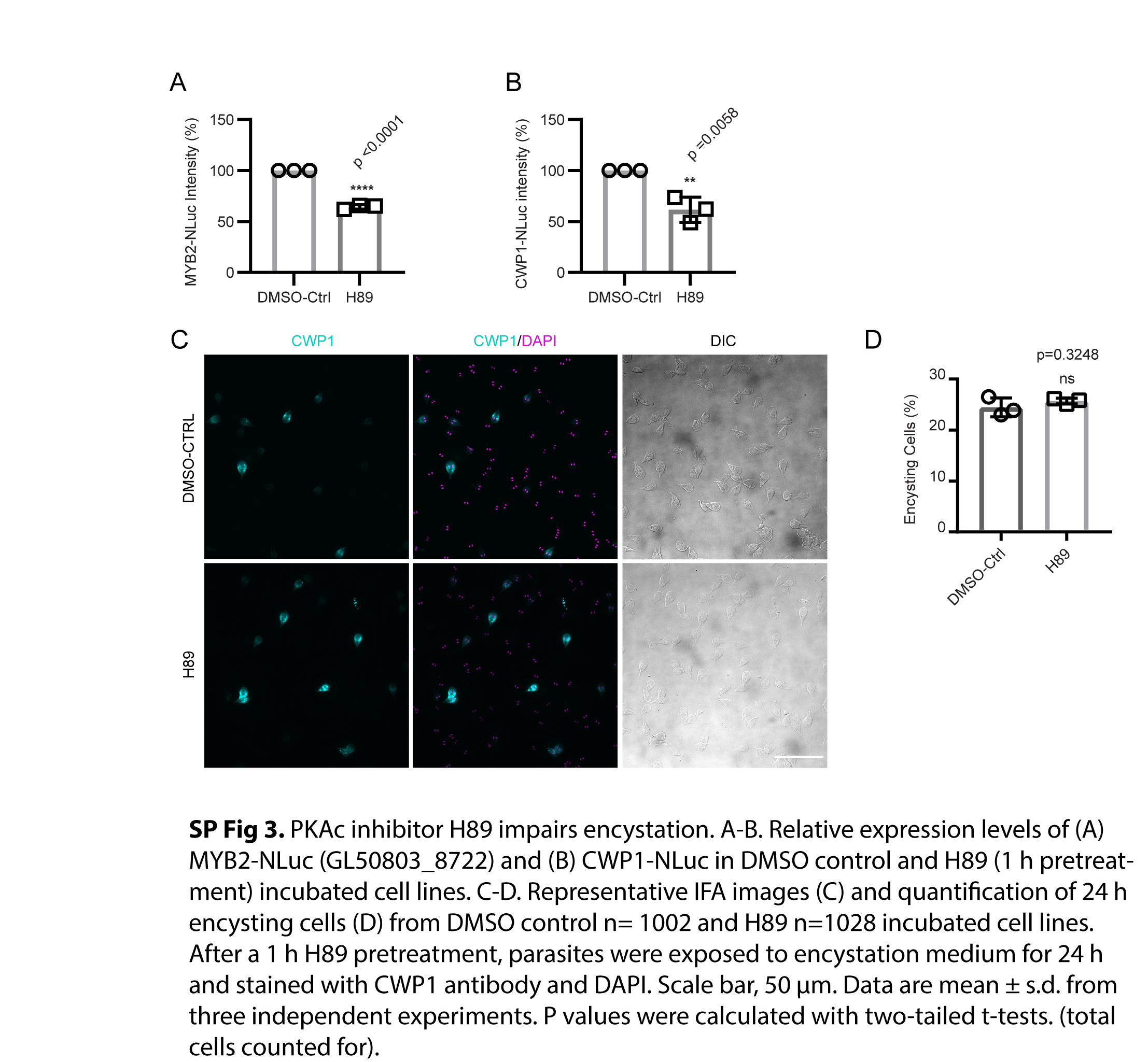

Supplement: Fig. S3 — PKAc inhibition with H89. [file msphere.00723-24-s0003.tif]
