# Supplementary material for: Unraveling the role of cAMP signaling in Giardia: insights into PKA-mediated regulation of encystation and subcellular interactions
Source: mSphere. 2024 Oct 30;9(11):e00723-24. doi: 10.1128/msphere.00723-24 (PMC11580427; doi:10.1128/msphere.00723-24)
Supplement: Table S1 — Oligos used in this study. [file msphere.00723-24-s0004.docx]

SP Table 1. Primers used in this study.

| Primer Name | Sequence (5’-3’) |
| --- | --- |
| PKAc-gRNA 378F | caaaGTAAATACCCCAATGCCAGG |
| PKAc-gRNA 378R | aaacCCTGGCATTGGGGTATTTAC |
| PKAc-gRNA 664F | caaaGGATTGTCATCGAAGAAGGG |
| PKAc-gRNA 664R | aaacCCCTTCTTCGATGACAATCC |
| PKAc-gRNA 704F | caaaGACCTGTAAGAAGATCCTTG |
| PKAc-gRNA 704R | aaacCAAGGATCTTCTTACAGGTC |
| PKAc-gRNA 848F | caaaGAAGCACGCATTCTTCTCAG |
| PKAc-gRNA 848R | aaacCTGAGAAGAATGCGTGCTTC |
| nHalo_for | GTCCATTCTTGAGCGGCCTAAAGCGcttaagatggcagaaatcggtactggctttc |
| nHalo_rev | gacgaccgagcaacgatgatgcaaagaattcTTAggtggtgcggaaggcctggaag |
| cHalo_for | AGACGCCCTGTTTGTGGGATTTAAAgtcgacgacgtcggccgcaagctgatcatcg |
| cHalo_rev | caaagctatccggctggaggatgctactagtttaaccggaaatctcgagcgtcgac |
